# Profiles and Outcomes of Women with Gestational Diabetes Mellitus in the United States

**DOI:** 10.7759/cureus.41360

**Published:** 2023-07-04

**Authors:** Oluwasegun A Akinyemi, Terhas Asfiha Weldeslase, Eunice Odusanya, Ngozi T Akueme, Ofure V Omokhodion, Mojisola E Fasokun, Deborah Makanjuola, Mary Fakorede, Temitayo Ogundipe

**Affiliations:** 1 Health Policy and Management, University of Maryland School of Public Health, College Park, USA; 2 Surgery, Howard University, Washington DC, USA; 3 Surgery, Howard University College of Medicine, Washington DC, USA; 4 Obstetrics and Gynecology, Howard University College of Medicine, Washington DC, USA; 5 Dermatology, University of Medical Sciences Teaching Hospital (UNIMEDTH), Ondo State, NGA; 6 Obstetrics and Gynecology, University College Hospital, Ibadan, NGA; 7 Epidemiology and Public Health, University of Alabama at Birmingham, Birmingham, USA; 8 Public Health, University of Alabama at Birmingham, Birmingham, USA; 9 Family Medicine, Howard University College of Medicine, Washington DC, USA; 10 Psychiatry, Ladoke Akintola University, Ogbomoso, NGA; 11 Community and Family Medicine, Howard University Hospital, Washington DC, USA

**Keywords:** epidermiology, united states, profile, fetal maternal outcomes, gestational diabetes mellitus (gdm)

## Abstract

Introduction

Gestational diabetes mellitus (GDM) is a major contributor to adverse pregnancy outcomes both in the United States and globally. As the prevalence of obesity continues to rise, the incidence of GDM is anticipated to increase as well. Despite the significant impact of GDM on maternal and neonatal health, research examining the independent associations between GDM and adverse outcomes remains limited in the U.S. context.

Objective

This study aims to address this knowledge gap and further elucidate the relationship between GDM and maternal and neonatal health outcomes.

Method

We performed a retrospective study using data from the United States Vital Statistics Records, encompassing deliveries that occurred between January 2015 and December 2019. Our analysis aimed to establish the independent association between GDM and various adverse maternal and neonatal outcomes. The multivariate analysis incorporated factors such as maternal socioeconomic demographics, preexisting comorbidities, and conditions during pregnancy to account for potential confounders and elucidate the relationship between GDM and the outcomes of interest.

Result

Between 2015 and 2019, there were 1,212,589 GDM-related deliveries, accounting for 6.3% of the 19,249,237 total deliveries during the study period. Among women with GDM, 46.4% were Non-Hispanic Whites, 11.4% were Non-Hispanic Blacks, 25.7% were Hispanics, and 16.5% belonged to other racial/ethnic groups. The median age of women with GDM was 31 years, with an interquartile range of 27-35 years. The cesarean section rate among these women was 46.5%. GDM was identified as an independent predictor of adverse maternal and neonatal outcomes, including cesarean section (OR=1.40; 95% CI: 1.39-1.40), maternal blood transfusion (OR=1.15; 95% CI: 1.12-1.18), intensive care unit admission (OR=1.16; 95% CI: 1.10-1.21), neonatal intensive care unit admission (OR=1.53; 95% CI: 1.52-1.54), assisted ventilation (OR=1.37; 95% CI: 1.35-1.39), and low 5-minute Apgar score (OR=1.01; 95% CI: 1.00-1.03).

Conclusion

GDM serves as an independent risk factor for adverse maternal and neonatal outcomes, emphasizing the importance of early detection and management in pregnant women.

## Introduction

Gestational diabetes mellitus (GDM) is a significant factor in the occurrence of unfavorable pregnancy outcomes both on a global scale and within the United States. It is a prevalent metabolic disorder identified during pregnancy and affects women who do not possess a prior medical record of diabetes [1]. Typically emerging around the 24th week of gestation, GDM arises from impaired glucose tolerance due to placental hormones interfering with maternal insulin regulation [2,3]. Women with a history of GDM face an increased risk of future cardiovascular disease, type 2 diabetes mellitus, and chronic kidney disease [4,5]. Additionally, GDM is associated with higher incidences of pre-eclampsia, cesarean delivery, polyhydramnios, stillbirth, and extended maternal hospitalization [1,6]. The consequences of GDM also extend to offspring, with infants born to mothers with poorly controlled blood sugar experiencing macrosomia, preterm birth, hypoglycemia, congenital abnormalities, obesity, diabetes, respiratory distress syndrome, and perinatal mortality [1,7]. Risk factors for GDM include maternal age (>25 years old), overweight/obesity, physical inactivity, polycystic ovary syndrome (PCOS), previous GDM, or prediabetes [3,7]. Despite increased awareness and early screening efforts, the prevalence of GDM continues to rise worldwide and in the United States [8].

The International Diabetes Federation (IDF) estimated in 2017 that 14% of pregnancies worldwide, or approximately 18.4 million pregnancies, were affected by GDM [9]. By 2019, one in six births involved women with GDM [10]. In the United States, the Centers for Disease Control and Prevention (CDC) reported that 2% to 10% of all pregnant women are diagnosed with GDM annually [2]. From 2016 to 2020, the rate of GDM increased by 30%, with 7.8 GDM cases per 100 births in 2020 compared to 6.0 GDM cases per 100 births in 2016 [4]. The economic burden of GDM in the United States has also risen dramatically, increasing from nearly $596 million for mothers and $40 million for newborns in 2007 to a total of $1.6 billion in 2017, doubling within a decade [11,12].

Existing literature reveals disparities in the prevalence of GDM among different populations. In the United States, Non-Hispanic Asian mothers experience the highest rate of GDM, with approximately 14.9 cases per 100 births [4]. Although non-Hispanic Black women have a lower risk of developing GDM (6.5 per 100 births), they are approximately 10 times more likely to develop type 2 diabetes following GDM [4,13]. The 2020 GDM rate for mothers under age 20 was approximately 2.5%, substantially lower than the 15.3% rate observed among those aged 40 and above [4]. Additionally, women residing in low-income communities are disproportionately affected by GDM, primarily due to limited access to affordable and reliable healthcare services [14].

Given the limited research on the independent association between GDM and adverse maternal and neonatal outcomes in the United States, this study aims to investigate the specific impact of GDM, irrespective of maternal socioeconomic status. By conducting a retrospective analysis of deliveries involving women with GDM in the United States, we intend to establish profiles of women with GDM and assess GDM as a primary predictor of adverse maternal and neonatal outcomes.

## Materials and methods

We obtained the data for this study from the United States Vital Statistics Records for 2015-2019. The National Vital Statistics Records database contains comprehensive information on births and deaths in the United States [12]. As the database is de-identified and publicly accessible, ethical clearance or Institutional Review Board approval was not required.

Study population

Our analysis included 19,249,237 deliveries documented in the United States Vital Statistics records between January 2015 and December 2019. The study cohort encompassed women of all races/ethnicities. We excluded participants with missing critical data, such as maternal race/ethnicity, body mass index (BMI), and age, and evaluated pregnancy outcomes from the study.

Definition of study outcomes

In this study, we aimed to determine the independent association between women's GDM status and selected primary measures of pregnancy outcomes. The maternal outcomes assessed were cesarean section, mothers requiring blood transfusion during labor or delivery (maternal blood transfusion), and maternal admission to the intensive care unit (maternal ICU admissions). The neonatal outcomes evaluated included a low 5-minute Apgar score (defined as Apgar score < 7), neonatal intensive care unit (NICU) admissions, and neonates receiving assisted ventilation for ≥6 hours after delivery.

Patient characteristics and risk factors

We selected covariates for this study based on existing literature, encompassing maternal and paternal demographics as well as obstetric and medical history. These factors included maternal race/ethnicity, classified as non-Hispanic White (White), non-Hispanic Black (Black), Hispanic, and non-Hispanic other (other), maternal insurance types (private insurance, public insurance (Medicaid), and uninsured), and maternal education level defined as high school, college, or advanced education. Additional factors incorporated in the study were maternal age, paternal race/ethnicity, paternal age, paternal education level, pre-pregnancy diabetes, hypertension, pre-pregnancy obesity, cesarean section and previous cesarean section (PCS), augmentation and labor induction, and delivery weight.

Statistical analysis

Categorical variables were expressed as frequencies and percentages. The Pearson chi-square test was used to compare categorical variables. Bivariate logistic models were used to calculate unadjusted odds ratios (ORs) and 95% CIs for the association of study variables with women’s GDM status. In cases of significant association, a post-hoc analysis was conducted between all groups to identify the source of statistical significance.

The final multivariate analysis was adjusted for maternal age, race/ethnicity, education, insurance types, pre-pregnancy diabetes, chronic hypertension, weight gain in pregnancy, PCS, induction of labor, delivery weight, and gestational age at delivery. We then determined the association between women’s GDM status and selected measures of maternal and neonatal outcomes. A two-tailed p-value of <0.05 was considered statistically significant. All statistical analyses were performed using STATA 16 (StataCorp, College Station, TX).

## Results

Table 1 presents the baseline characteristics of the study population stratified by GDM status. Of the 19,249,237 million deliveries, 51.4% were non-Hispanic whites, 24.0% Hispanics, and 14.3% non-Hispanic blacks. Although non-Hispanic whites predominated in the total population and GDM subpopulation, the proportion of non-Hispanic blacks among GDM patients (11.4%) was lower than in the total population (14.3%).

**Table 1:** Baseline demographics, risk factors, and outcomes stratified by race/ethnicity

Variables	Total Population	GDM	No GDM	p-Value
	(N=19,249,237)	(n=1,212,589)	(n=18,036,648)	
Race/ethnicity				<0.001
Non-Hispanic whites	9,892,500 (51.4%)	562,623 (46.1%)	9,329,877 (51.7%)	
Non-Hispanic blacks	2,742,363 (14.3%)	138,008 (11.4%)	2,604,355 (14.4%)	
Hispanics	4,615,897 (24.0%)	311,683 (25.7%)	4,304,214 (23.8%)	
Non-Hispanic American Indian/Alaska Native	148,306 (0.8%)	14,036 (1.2%)	134,270 (0.7%)	
Non-Hispanic Asian/Pacific Islander	1,222,184 (6.4%)	146,100 (12.0%)	1,076,084 (6.0%)	
Non-Hispanic others	57,721 (0.3%)	4,610 (0.4%)	53,111 (0.3%)	
Non-Hispanic mixed race	404,592 (2.1%)	24,542 (2.0%)	380,050 (2.1%)	
Unknown	160,040 (0.8%)	10,760 (0.9%)	149,280 (0.8%)	
Age (years)				<0.001
≤19	999,242 (5.2%)	20,539 (1.7%)	978,703 (5.4%)	
20-24	3,849,333 (20.0%)	132,368 (10.9%)	3,716,965 (20.6%)	
25-29	5,579,992 (29.0%)	298,270 (24.6%)	5,281,722 (29.3%)	
30-34	5,444,285 (28.3%)	402,243 (33.2%)	5,042,042 (28.0%)	
35-39	2,751,078 (14.3%)	276,648 (22.8%)	2,474,430 (13.7%)	
≥40	619,673 (3.2%)	82,294 (6.8%)	537,379 (3.0%)	
Education				<0.001
Elementary	2,521,226 (13.3%)	161,598 (13.5%)	2,359,628 (13.3%)	
Pre-college	8,730,947 (45.9%)	528,001 (44.1%)	8,202,946 (46.1%)	
Tertiary	5,469,446 (28.8%)	358,893 (30.0%)	5,110,553 (28.7%)	
Postgraduate	2,284,856 (12.0%)	147,734 (12.4%)	2,137,122 (12.0%)	
Insurance				<0.001
Medicaid	8,155,056 (42.6%)	502,245 (41.6%)	7,652,811 (42.7%)	
Private	9,430,667 (49.3%)	622,306 (51.6%)	8,808,361 (49.1%)	
Self-pay	810,031 (4.2%)	38,389 (3.2%)	771,642 (4.3%)	
Other	734,066 (3.8%)	43,181 (3.6%)	690,885 (3.9%)	
BMI				<0.001
Underweight	631,751(3.4%)	18,624 (1.6%)	613,127 (3.5%)	
Normal	8,112,616 (43.2%)	305,522 (25.8%)	7,807,094 (44.4%)	
Overweight	4,944,021 (26.3%)	312,831 (26.4%)	4,631,190 (26.3%)	
Obese class I	2,765,555 (14.7%)	253,771 (21.4%)	2,511,784 (14.3%)	
Obese class II	1,356,161 (7.2%)	157,447 (13.3%)	1,198,714 (6.8%)	
Obese class III	964,854 (5.1%)	137,782 (11.6%)	827,072 (4.7%)	
Weight gain (grams)				<0.001
<11	1,789,968 (9.6%)	197,363 (16.8%)	1,592,605 (9.1%)	
11-20	3,238,566 (17.4%)	278,554 (23.7%)	2,960,012 (17.0%)	
21-30	5,275,252 (28.3%)	320,344 (27.2%)	4,954,908 (28.4%)	
31-40	4,515,185 (24.3%)	207,399 (17.3%)	4,307,786 (24.7%)	
≥41	3,803,019 (20.4%)	172,519 (14.7%)	3,630,500 (20.8%)	
Birth weight (kg)				<0.001
<2,500	1,569,431 (8.2%)	109,929 (9.1%)	1,459,502 (8.1%)	
2,500-3,999	16,168,597 (84.1%)	978,435 (80.7%)	15,190,162 (84.3%)	
4,000-4,499	1,292,826 (6.7%)	98,765 (8.2%)	1,194,061 (6.6%)	
≥4,500	200,845 (1.0%)	24,880 (2.1%)	175,965 (1.0%)	
Prediabetes	173,297 (0.9%)	0 (0.0%)	173,297 (1.0%)	<0.001
Chronic hypertension	366,503 (1.9%)	55,235 (4.6%)	311,268 (1.7%)	<0.001
Previous cesarean section	2,978,278 (14.5%)	252,616 (20.8%)	2,725,662 (15.1%)	<0.001
Breastfeeding	13,765,790 (82.2%)	869,468 (82.6%)	12,896,322 (82,1%)	<0.001
Induction of labor	5,012,592 (26.0%)	423,943 (35.0%)	4,588,649 (25.5%)	<0.001
Augmentation of labor	4,096,123 (21.3%)	232,002 (19.1%)	3,864,121 (21.4%)	<0.001

GDM patients tended to be older, with 62.8% aged 30 or above, compared to 54.2% in the total population. The proportion of women with a minimum college education was similar between the total population and GDM patients (approximately 40%). Notably, 51.6% of GDM patients utilized private insurance compared to 49.3% in the total population.

The prevalence of higher BMI increased among GDM patients, contrary to the trend observed in the total population. As weight gain increased, GDM incidence significantly decreased. A slightly higher proportion of underweight and fetal macrosomia was observed among GDM patients.

Interestingly, no GDM patients reported a previous history of diabetes, while 0.9% of the total population and 1.0% of women without GDM did. GDM patients exhibited a higher incidence of PCSs. They were more likely to undergo labor induction but had a slightly lower incidence of labor augmentation than the total population. Breastfeeding rates did not differ between the groups.

Table 2 presents the unadjusted associations between selected maternal and neonatal outcomes and GDM status. Women with GDM experienced a higher cesarean section rate (42.6% vs. 31.3%) and higher maternal transfusion rate (0.5% vs. 0.4%) compared to the reference group of women without GDM. Additionally, the likelihood of ICU admission was greater for GDM patients (0.24% vs. 0.16%).

**Table 2:** Unadjusted association between GDM status and selected maternal and neonatal outcomes GDM, gestational diabetes mellitus; ICU, intensive care unit; SCBU, special care baby unit

Variable	Total	GDM	NO GDM	p-Value
	(N=1,92,49,237)	(n=12,12,589)	(n=1,80,36,648)	
Cesarean section	61,66,283 (32.0%)	5,16,369 (42.6%)	56,49,914 (31.3%)	<0.001
Maternal transfusion	73,848 (0.40%)	5,997 (0.50%)	67,851 (0.4%)	<0.001
ICU admission	31,441 (0.16%)	2,858 (0.24%)	28,583 (0.16%)	<0.001
5-minute Apgar	3,86,687 (2.0%)	25,906 (2.1%)	3,60,781 (2.0%)	<0.001
SCBU admission	17,08,039 (8.9%)	1,57,797 (13.0%)	15,50,242 (8.6%)	<0.001
Assisted ventilation	2,78,157 (1.5%)	26,234 (2.2%)	2,51,923 (1.4%)	<0.001

Neonates born to mothers with GDM exhibited a marginally higher incidence of low 5-minute Apgar scores (2.1% vs. 2.0%). Although statistically significant, this difference may not be clinically meaningful. Furthermore, these neonates demonstrated a higher rate of special care baby unit (SCBU) admissions (13.0% vs. 8.6%) compared to those born to mothers without GDM. The elevated SCBU admission rate could be attributed to an increased risk of complications, such as prematurity, hypoglycemia, hypocalcemia, and other electrolyte imbalances or congenital malformations.

**Figure 1:**
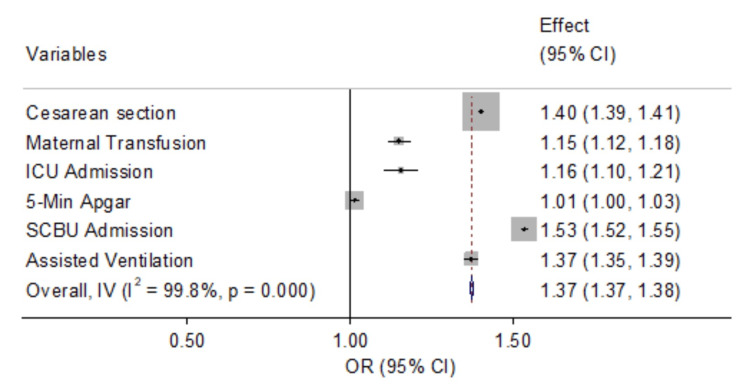
Independent association between GDM status and selected maternal and neonatal outcomes GDM, gestational diabetes mellitus; ICU, intensive care unit; SCBU, special care baby unit

Figure 1 displays the independent associations between GDM and selected maternal and neonatal outcomes. Women with GDM have a 1.40-fold increased likelihood of undergoing a cesarean section compared to the reference group. Additionally, these women exhibit a 15% higher odds of requiring blood transfusion during the index pregnancy, indicating an elevated risk of both antenatal and postpartum hemorrhage. Consequently, it is not surprising that women with GDM experience an almost 16% increased risk of ICU admission during pregnancy. Neonates born to mothers with GDM demonstrate a slightly elevated risk of low 5-minute Apgar scores (defined as an Apgar score < 7).

## Discussion

This study analyzed 19,249,237 million deliveries to investigate the associations between GDM and various maternal and neonatal factors. Our findings reveal that non-Hispanic whites constituted most of the total population and the GDM subpopulation. However, it is important to interpret this with caution as this may simply reflect the higher proportion of non-Hispanic whites in the total population of this study since a higher prevalence of GDM among minority populations has been documented in the literature, reinforcing disparities in GDM prevalence among racial and ethnic groups [15].

We observed that GDM patients tended to be older, consistent with previous research indicating that advanced maternal age is a risk factor for GDM [16]. GDM risk increases with maternal age due to age-related decline in insulin sensitivity, increased prevalence of obesity, and altered hormonal balance [16]. The proportion of women with a minimum college education was similar between the total population and GDM patients. Education may influence GDM risk indirectly. Higher education levels are often linked with healthier lifestyles, including a balanced diet and regular exercise, and reducing obesity risk, a key GDM risk factor. Additionally, educated individuals might have better healthcare access and health literacy, enabling early GDM detection and management. A slightly higher percentage of GDM patients utilized private insurance.

Our study found the highest prevalence of GDM among obese women, corroborating existing literature on the relationship between BMI and GDM risk [17]. This association may be due to insulin resistance caused by higher BMI and lifestyle factors. Interestingly, we noted a significant decrease in GDM incidence with increased weight gain in pregnancy. This counter-intuitive trend might suggest metabolic adaptations or physiological changes in pregnancy aiding glucose regulation. However, this observation warrants a deeper inquiry, underscoring the need for further comprehensive research on this complex relationship. In addition, GDM patients displayed a slightly higher proportion of underweight and fetal macrosomia, which has been associated with adverse perinatal outcomes [18].

None of the GDM patients reported a previous history of diabetes, while a small percentage of the total population and women without GDM did. This finding suggests a potential underreporting of pre-existing diabetes among GDM patients. GDM patients exhibited a higher incidence of PCSs. They were more likely to undergo labor induction but had a slightly lower incidence of labor augmentation than the total population. Breastfeeding rates were similar between the groups.

These findings highlight the importance of understanding demographic, clinical, and lifestyle factors associated with GDM to optimize prenatal care and improve maternal and neonatal outcomes [19]. Healthcare providers should consider these factors when developing targeted interventions for preventing and managing GDM. Further research is needed to explore potential causal pathways and the long-term effects of GDM on maternal and neonatal health.

Our findings also reveal that women with GDM are more likely to undergo a cesarean section than the reference group, consistent with prior research [20]. This increased risk may be attributed to various factors, including fetal macrosomia and higher rates of labor induction and augmentation among GDM patients [21].

Additionally, women with GDM have higher odds of requiring blood transfusion during the index pregnancy, indicating an elevated risk of both antenatal and postpartum hemorrhage. This finding aligns with previous studies suggesting that women with GDM have a greater risk of experiencing hemorrhage during delivery [22]. Subsequently, these women have a higher risk of needing a blood transfusion, reinforcing the increased risk of antenatal and postpartum hemorrhage. The literature suggests this could be due to higher rates of operative deliveries (cesarean sections) and placental abnormalities related to GDM. Both factors can increase the likelihood of severe bleeding, necessitating blood transfusions [22].

Our results also demonstrate that women with GDM experience an increased risk of ICU admission during pregnancy. This increased risk may be attributed to GDM-related complications such as hypertension, preeclampsia, and other comorbidities [23].

Neonates born to mothers with GDM demonstrate a slightly elevated risk of low 5-minute Apgar scores (defined as an Apgar score < 7). This finding is consistent with prior research that has reported an association between GDM and adverse neonatal outcomes, including low Apgar scores [24]. For several reasons, GDM increases the risk of low 5-minute Apgar scores in neonates. Maternal hyperglycemia can lead to fetal hyperinsulinemia, potentially causing birth complications. GDM can also result in macrosomia, leading to difficult deliveries. Additionally, GDM heightens the likelihood of preterm birth and neonatal hypoglycemia, which can contribute to lower Apgar scores. Furthermore, respiratory distress syndrome, more common in babies born to mothers with GDM, can negatively affect Apgar scores. These factors highlight the necessity of close monitoring during pregnancies complicated by GDM.

After adjusting for known traditional risk factors, neonates born to mothers with GDM exhibit a significantly increased likelihood of SCBU admission and also a higher risk of requiring assisted ventilation for more than 6 hours post-delivery. Neonates born to mothers with GDM have a heightened risk of requiring special care and assisted ventilation. This is likely due to complications such as neonatal hypoglycemia, respiratory distress syndrome, and macrosomia, which are more prevalent among babies born to mothers with GDM, as per existing literature.

Across all assessed maternal and neonatal outcomes, GDM consistently emerges as an independent predictor of adverse pregnancy outcomes.

Limitations

This study identified several key associations between GDM and adverse maternal and neonatal outcomes. However, it is important to acknowledge certain limitations that may impact the interpretation and generalizability of our findings.

Retrospective Design

Our study utilized a retrospective design, which inherently limits our ability to infer causality. Prospective, longitudinal studies would be better suited to establish causal relationships between GDM and the observed outcomes.

Data Quality and Completeness

As with any large-scale database study, data quality and completeness could be a potential concern. Missing or inaccurate data may affect the validity of our results.

Confounding Factors

Although we adjusted for several known risk factors, residual confounding may still exist due to unmeasured or unknown variables. Additionally, our study did not explore potential effect modification by factors such as race/ethnicity, socioeconomic status, or maternal health behaviors, which may influence the relationships between GDM and the examined outcomes.

Generalizability

The study population may not be representative of all pregnant women, limiting the generalizability of our findings to other populations or healthcare settings.

Temporal Changes in GDM Management

Our analysis is based on data from a specific period, and GDM management strategies may have evolved since then. Consequently, our findings may not accurately reflect the current state of GDM-related outcomes.

## Conclusions

In conclusion, our study demonstrates significant associations between GDM and increased risks of adverse maternal and neonatal outcomes. These findings underscore the importance of early identification and appropriate management of GDM in pregnant women to potentially reduce the incidence of complications for both mother and child.

Implications for future research include the need for prospective, longitudinal studies to establish causality and explore potential effect modifiers, such as race/ethnicity, socioeconomic status, and maternal health behaviors. Additionally, research should investigate the effectiveness of different GDM screening and management strategies to optimize patient outcomes. As GDM management continues to evolve, it is essential for future studies to stay up-to-date with the latest guidelines and protocols in order to provide accurate and relevant insights into the relationship between GDM and maternal and neonatal health. Ultimately, these investigations will contribute to the development of evidence-based interventions and policies that can improve the care of pregnant women with GDM and their offspring.
